# Study on the Influence Mechanism of Alkaline Earth Element Doping on the Thermoelectric Properties of ZnO

**DOI:** 10.3390/mi16080850

**Published:** 2025-07-24

**Authors:** Haitao Zhang, Bo Feng, Yonghong Chen, Peng Jin, Ruolin Ruan, Biyu Xu, Zhipeng Zheng, Guopeng Zhou, Yang Zhang, Kewei Wang, Yin Zhong, Yanhua Fan

**Affiliations:** 1 Institute of Engineering and Technology, Hubei University of Science and Technology, Xianning 437100, Chinawdufengbo@163.com (B.F.);; 2 Hubei Xiangcheng Intelligent Electromechanical Research Institute Co., Ltd., Xianning 437100, China; 3 Hubei MAGNIFICENT New Material Technology Co., Ltd., Xiangyang 441200, China; 4 Jiangsu MAGNIFICENT New Material Technology Co., Ltd., Yancheng 224000, China; 5 Shanghai Institute of Ceramics, Chinese Academy of Sciences, Shanghai 201899, China; 6 School of Electronic and Electrical Engineering, Wuhan Textile University, Wuhan 430200, China

**Keywords:** functional ceramics, density of states, conductivity, atomic hybridization, first principles

## Abstract

As a promising n-type semiconductor thermoelectric material, ZnO has great potential in the high-temperature working temperature range due to its advantages of abundant sources, low cost, high thermal stability, and good chemical stability, as well as being pollution-free. Sr-doped ZnO-based thermoelectric materials were prepared using the methods of room-temperature powder synthesis and high-temperature block synthesis. The phase composition, crystal structure, and thermoelectric performances of ZnO samples with different Sr doping levels were analyzed using XRD, material simulation software and thermoelectric testing devices, and the optimal doping concentrations were obtained. The results show that Sr doping could cause the Zn-O bond to become shorter; in addition, the hybridization between Zn and O atoms would become stronger, and the Sr atom would modify the density of states near the Fermi level, which could significantly increase the carrier concentration, electrical conductivity, and corresponding power factor. Sr doping could cause lattice distortion, enhance the phonon scattering effect, and decrease the lattice thermal conductivity and thermal conductivity. Sr doping can achieve the effect of improving electrical transport performance and decreasing thermal transport performance. The ***ZT*** value increased to ~0.418 at 873 K, which is ~4.2 times the highest ***ZT*** of the undoped ZnO sample. The Vickers hardness was increased to ~351.1 HV, which is 45% higher than the pristine ZnO.

## 1. Introduction

New energy can address the issues of the increasing depletion of fossil energy and the tight energy supply. The development and utilization of new energy are also essential for promoting the sustainable development of the energy industry. New energy includes solar energy, wind energy, geothermal energy, biomass energy, etc. Compared with fossil fuels, new energy has more abundant reserves, and its utilization is cleaner and more environmentally friendly, with less pollution to the environment. Among them, thermoelectric power generation technology can convert waste heat into electrical energy without transmission components, and it is currently one of the new energy technologies being vigorously developed [1,2,3]. Low-temperature power generation materials, such as Bi_2_Te_3_, have been commercially applied [4,5,6]. High-temperature power generation materials, such as PbTe, have been applied in military and aerospace fields [7,8,9]. However, due to cost and toxic elements, they are not universal and are not suitable for large-scale application. Therefore, the research on high-temperature power generation materials needs to be strengthened.

ZnO is the most promising high-temperature thermoelectric material. Compared with other high-temperature materials such as In_2_O_3_ [10,11] or SrTiO_3_ [12,13], ZnO is widely used in the electric and plastic industries and is a low-cost, non-toxic material with ease of manufacture. ZnO has emerged as the most promising high-temperature thermoelectric material, outshining other contenders in this domain. When contrasted with high-temperature materials like In_2_O_3_ and SrTiO_3_, ZnO brings a plethora of advantages that make it stand out in the race for efficient thermoelectric applications. In_2_O_3_, despite its research interest, has certain limitations. It often requires complex synthesis procedures and comes at a relatively higher cost. The manufacturing processes for In_2_O_3_-based materials can be intricate, involving multiple steps and precise control of conditions [14]. This not only adds to the production expenses but also poses challenges in scaling up the manufacturing process. SrTiO_3_, on the other hand, while having its own unique properties, also faces issues. Its application scope in some industries is restricted due to its specific chemical reactivity and physical characteristics. For example, in certain electric applications where compatibility with other materials is crucial, SrTiO_3_ might not be as adaptable as ZnO. In contrast, ZnO’s wide usage in the electric and plastic industries showcases its versatility. Zinc oxide is a staple in these sectors, boasting a long-established manufacturing base. Its cost-effectiveness represents a significant advantage: it can be produced in large quantities at a relatively low cost, rendering it accessible for a wide range of applications. Furthermore, its non-toxicity is a major plus, particularly in applications where environmental and health concerns are of utmost importance. In the plastic industry, for instance, the use of non-toxic zinc oxide ensures that plastic products are safe for consumer use. As a Ⅱ-Ⅵ semiconductor material, ZnO has a wide band gap, measuring above 3 eV [15,16]. This wide band gap endows ZnO with distinct electrical and optical properties. The nature of the chemical bond in ZnO, which lies in the transition zone between an ionic bond and a covalent bond, plays a vital role in determining its material characteristics. The ionic–covalent hybrid bond contributes to the stability of the ZnO structure and also affects the movement of charge carriers within the material. The crystal structure of ZnO, belonging to the wurtzite structure of the hexagonal crystal family, further influences its properties. The wurtzite structure provides a specific arrangement of atoms, which affects the phonon dispersion and electron transport within the crystal [17]. With a melting point as high as ~2248 K and a free exciton binding energy up to 60 meV, ZnO is well-equipped to withstand high-temperature environments. These structural and band characteristics are the foundation for ZnO functional materials to exhibit a relatively high Seebeck coefficient, low carrier concentration, and high thermal conductivity [18,19]. The low carrier concentration, combined with a wide band gap, helps reduce unwanted electrical conduction that may lead to energy loss. However, while high thermal conductivity is beneficial in some respects, it also presents a challenge in thermoelectric applications. In thermoelectric devices, the goal is to maximize the conversion of heat into electricity, yet high thermal conductivity can cause heat to flow through the material without being efficiently converted into electrical energy [20]. To enhance the thermoelectric performance of ZnO, two key aspects need to be addressed: improving electrical conductivity and reducing thermal conductivity. Numerous research efforts have focused on doping the Zn site, and the principle behind this approach is based on defect chemistry [21]. By introducing dopants, the carrier concentration and electrical conductivity of ZnO can be increased. In addition to these single-element dopings, there is also growing interest in co-doping or multi-doping ZnO. Co-doping involves introducing two or more different dopants simultaneously. This approach can lead to synergistic effects. For example, one dopant might be effective in increasing the carrier concentration, while another can help in reducing the thermal conductivity. By carefully selecting the combination and concentration of dopants, the thermoelectric properties of ZnO can be optimized more effectively. Another research direction focuses on the nanostructuring of ZnO—by creating nanostructures such as nanowires, nanorods, or nanoporous structures, phonon scattering can be enhanced. Phonons are the carriers of heat in solids; enhancing phonon scattering can reduce thermal conductivity. Meanwhile, nanostructuring also affects electrical transport properties, potentially improving the overall thermoelectric figure of merit. Furthermore, research on the interface properties of ZnO-based composites is becoming increasingly important: when ZnO is combined with other materials to form composites, the interfaces between ZnO and other components play a crucial role in determining thermoelectric performance. These interfaces can act as barriers for phonons to reduce thermal conductivity, while also facilitating the transfer of charge carriers to enhance electrical conductivity. Understanding and controlling these interface properties through techniques like surface modification and interface engineering can open up new avenues for improving the thermoelectric performance of ZnO-based materials. The development of new synthesis methods for ZnO is also an active area of research. Conventional synthesis methods have their limitations, and new techniques are being explored to produce ZnO with more precise control over its structure, composition, and defect states. For example, hydrothermal synthesis can produce ZnO nanomaterials with well-defined shapes and sizes. This method enables the control of reaction conditions such as temperature, pressure, and pH, which can affect the properties of the resulting ZnO. Another emerging approach is atomic layer deposition, which allows for the deposition of ZnO films with atomic-level precision. Such precise control over film growth can lead to ZnO materials with enhanced thermoelectric properties. To improve its thermoelectric performance, it is necessary to enhance its electrical conductivity and reduce its thermal conductivity. Many studies have doped the Zn site to increase carrier concentration and electrical conductivity through defect chemistry principles, such as Al doping [22,23], Sn doping [24], Ni doping [25,26], Sm doping [27], Y doping [28], etc. When Al is doped into ZnO, it can substitute Zn atoms in the crystal lattice. Al has one more valence electron than Zn, and this extra electron can be easily excited into the conduction band, increasing the number of charge carriers and thus enhancing electrical conductivity. Similarly, Sn doping can introduce additional electrons into the system. The size and electronic configuration of Sn atoms enable them to fit into the ZnO lattice, thereby contributing to electrical conduction. Ni doping in ZnO has shown interesting results: Ni atoms can occupy Zn sites and modify the local electronic structure, which in turn leads to changes in the band structure of ZnO, creating new energy levels that facilitate electron movement. On the other hand, Sm doping has been found to influence the grain growth and microstructure of ZnO; by altering the microstructure, both electrical and thermal transport properties can be tuned. Studies on Y doping aim to understand its effect on the crystal structure and defect formation in ZnO. With their specific ionic radius and electronic properties, Y atoms can interact with the ZnO lattice, thereby affecting the overall thermoelectric performance.

The basic reason for the low electrical properties of ZnO is the wide band gap and the low density of states near the Fermi level. Here, Sr, an alkaline earth element with rich electronic structure, is used to dope ZnO at Zn site in order to modify the electronic band structure and improve the thermoelectric performances. The electronic band structure of alkaline earth elements exhibits high density of states, which is conducive to modifying the density of states near the Fermi level when used to dope oxides, thus improving the electrical transport performance [29,30,31,32]. The innovations of Sr-doped zinc oxide thermoelectric materials lie mainly in two key aspects. Firstly, as an alkaline earth metal element, Sr has a uniquely rich electronic structure. With an outer electron configuration of 5s^2^, compared to Zn’s 3d^10^4s^2^, Sr can more easily form impurity energy levels through electron transitions during doping. These impurity levels can effectively adjust the carrier concentration of zinc oxide, providing additional channels for electron transport, thereby significantly improving the material’s electrical transport performance—it can not only optimize electrical conductivity but also maintain a high Seebeck coefficient within a certain range, solving the problem of low efficiency when relying solely on intrinsic defects to regulate carriers. Secondly, there is a difference in ionic radius between Sr^2+^ (118 pm) and Zn^2+^ (74 pm). When Sr^2+^ replaces Zn^2+^ in the lattice, it causes significant lattice distortion. This distortion can not only enhance phonon scattering, effectively reducing lattice thermal conductivity by scattering medium- and high-frequency phonons, but also precisely regulate carrier mobility. More importantly, it breaks the “trade-off relationship” between electrical conductivity and Seebeck coefficient in traditional doping (i.e., the increase in electrical conductivity is often accompanied by a decrease in Seebeck coefficient), achieving their synergistic optimization and providing a new mechanism for performance breakthroughs of zinc oxide-based thermoelectric materials in the medium- and high-temperature fields. The first principles were used here to calculate the energy bands and density of states before and after Sr doping with ZnO, and experimental testing was conducted to verify the electrical transport performance parameters. The optimization mechanism of the thermoelectric performance of ZnO functional materials was explored through a combination of simulation calculation and experimental testing.

## 2. Experimental Part

Here, a series of samples of Zn_1−*x*_Sr*_x_*O (*x* = 0, 0.005, 0.010, 0.015, 0.020, 0.025) are prepared by first making powder and then making blocks. The first step is to weigh ZnO and SrO powder samples (Sinopharm, 99.99%) in the Glovebox according to the stoichiometric ratio. The second step is to perform mechanical alloying high-energy ball milling (including ball milling in a protective atmosphere and ball milling with alcohol, for a total of 13 h). The third step is to dry the obtained powder for more than 48 h to remove moisture. The fourth step is to perform spark plasma sintering on the dried powder obtained (sintering temperature is 1273 K, sintering pressure is 60 MPa).The fifth step is to cut the obtained block sample and test parameters such as XRD, electrical conductivity, Seebeck coefficient, and thermal conductivity. We calculate the energy bands and density of states of materials based on computational materials science using Materials Studio software (8.0). The specific experimental process parameters, equipment models, and simulation methods can be found in the Supporting Materials [33], enabling other researchers to reproduce the experiment and build on the findings.

## 3. Results and Discussion

**Figure 1:**
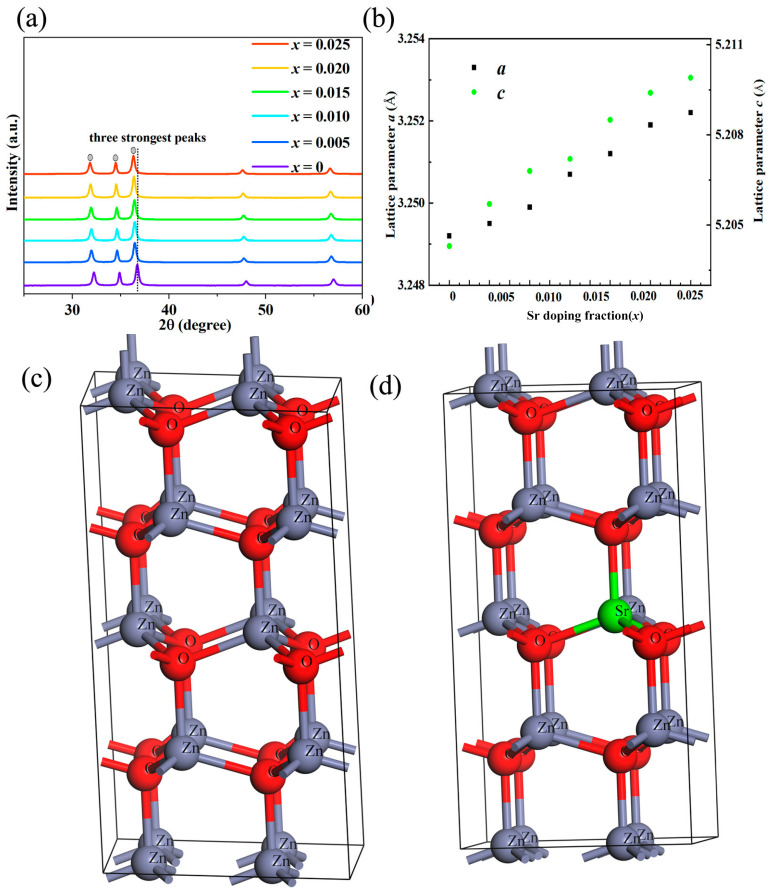
(**a**) The XRD patterns; (**b**) the lattice constant of Sr-doped ZnO series polycrystalline samples; the crystal structure model of ZnO (**c**) and Sr-doped ZnO (**d**).

Figure 1 shows the bulk XRD pattern of Sr-doped ZnO samples. The XRD diffraction peaks of all samples are consistent with the diffraction peaks of ZnO (the PDF card for XRD detection of zinc oxide thermoelectric materials is #36-1451, which corresponds to zinc oxide with a hexagonal wurtzite structure), and no second phase is found in the samples after Sr doping. It is worth noting that the lattice constants (***a*** and ***c***) of the doped sample increase slightly compared with the undoped ZnO to a certain extent, which can be attributed to the difference in the ionic radius of the doped element and the substituted element (the ionic radius of Sr^2+^ (~1.18 Å) is larger than the ionic radius of Zn^2+^ (~0.74 Å)). The crystallography data as shown in Table 1 before and after Sr doping, calculated by the first-principle method, are consistent with it. The lattice expansion caused by Sr doping should be due to Vegard’s law [34,35].

**Figure 2:**
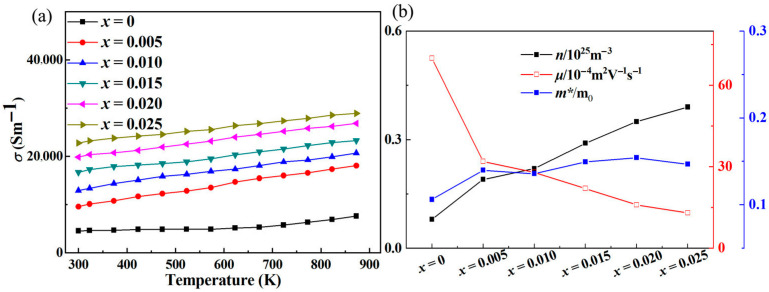
(**a**) The relationship between the electrical conductivity (***σ***) of the samples and the measured temperature; (**b**) the relationship between carrier concentration (***n***), mobility (***μ***), effective mass (m*/m_0_) and the doping content of Sr.

**Figure 3:**
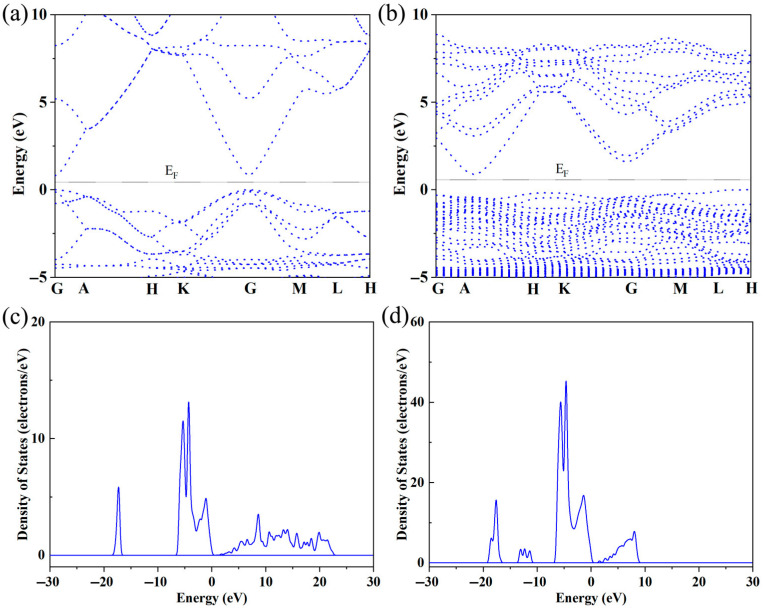
(**a**) The band structure of ZnO; (**b**) the band structure of Sr-doped ZnO; (**c**) the DOS of ZnO; (**d**) the DOS of Sr-doped ZnO.

**Figure 4:**
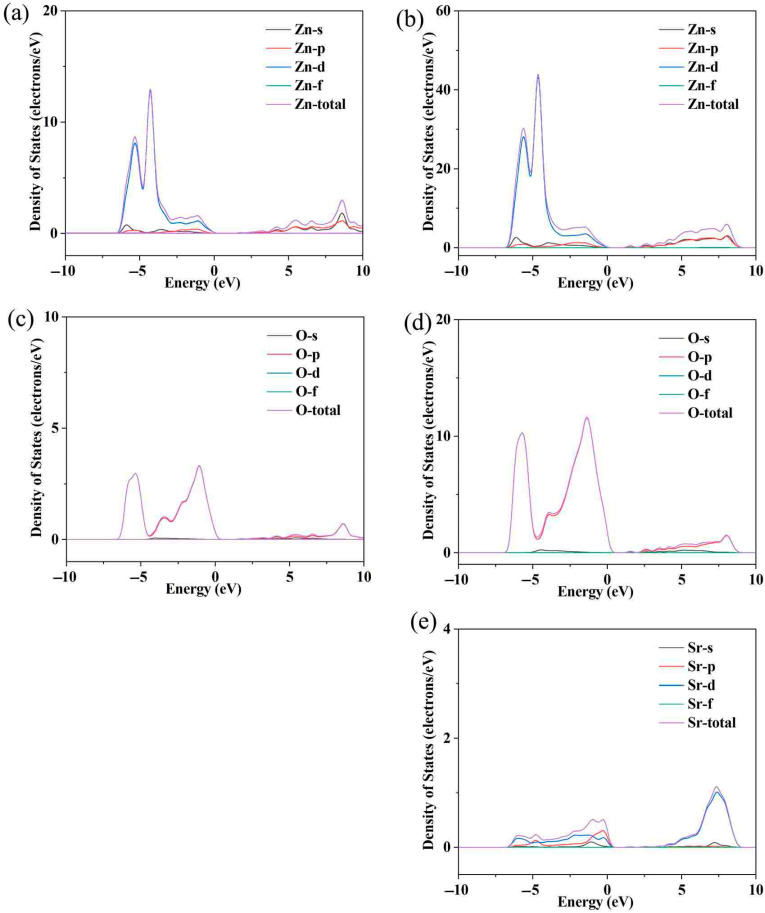
The partial densities of states (PDOS) for Zn atoms of ZnO (**a**) and Sr-doped ZnO (**b**); the partial densities of states (PDOS) for O atoms of ZnO (**c**) and Si-doped ZnO (**d**); the partial densities of states (PDOS) for Sr atoms in Sr-doped ZnO (**e**); the schematic diagram of band convergence caused by Sr doping.

Figure 2a shows the variation in electrical conductivity (*σ*) with temperature in Sr-doped samples. In order to explore the reasons for the increase in conductivity more clearly, we also provide the relationship between carrier concentration (***n***) and carrier mobility (***μ***) with temperature through the Hall testing system in Figure 2b. The undoped ZnO sample exhibits low conductivity throughout the entire testing temperature range, with a maximum of only ~75 Scm^−1^. This is due to the wide band gap and low carrier concentration limitations of ZnO itself. After Sr doping, the carrier concentration and corresponding conductivity significantly increased throughout the entire testing temperature range, reaching a maximum of ~289 Scm^−1^, which is ~3.85 times that of the pristine one. Considering that both Sr and Zn exhibit + 2 valence, the reason for the increase in carrier concentration is not due to donor effects [36,37]. In order to further investigate the reasons for the increase in carrier concentration and conductivity, first principles were used to establish an atomic model and calculate the energy band of ZnO before and after Sr doping. The calculated electronic band structure and density of states are shown in Figure 3. The density of states’ enhancement effect can enable more electrons to participate in the transport, thus improving the carrier concentration and conductivity. In order to further analyze the density of states near the Fermi level, the CASTEP module was used to calculate the partial density of states (PDOS) of each atom before and after Sr doping, as shown in Figure 4. After Sr doping, the density of states of Zn atom and O atom near the Fermi energy level is significantly modified. After Sr doping, the Zn-O bond becomes shorter (as shown in Table 1), which makes the electronic orbits of Zn and O have more overlap and hybridization [38]. The hybridization between Sr and Zn/O can cause changes in microscopic results such as bond length and bond angle within the zinc oxide matrix, corresponding to changes in electron and phonon transport. The state density of the Sr atom itself is not high—the highest reaches ~2.8 electrons/eV—and the Sr-O bond is shorter than the Zn-O bond, as shown in Table 1. The hybridization enhancement of Zn and O atoms caused by Sr doping is the main reason for the change in state density near the Fermi level. Although the changes in the DOS of ZnO after Sr doping do not clearly support improved electronic transport properties and the band gap increased, the carrier concentration and electrical conductivity increased according to the testing results. This may be because Sr doping shortens the Zn-O bond length and enhances the hybridization between Zn and O atomic orbitals. Stronger hybridization promotes electron delocalization and improves electronic transport properties. Meanwhile, carriers introduced by Sr doping compensate for the adverse effect of increased band gap, ultimately increasing conductivity.

**Figure 5:**
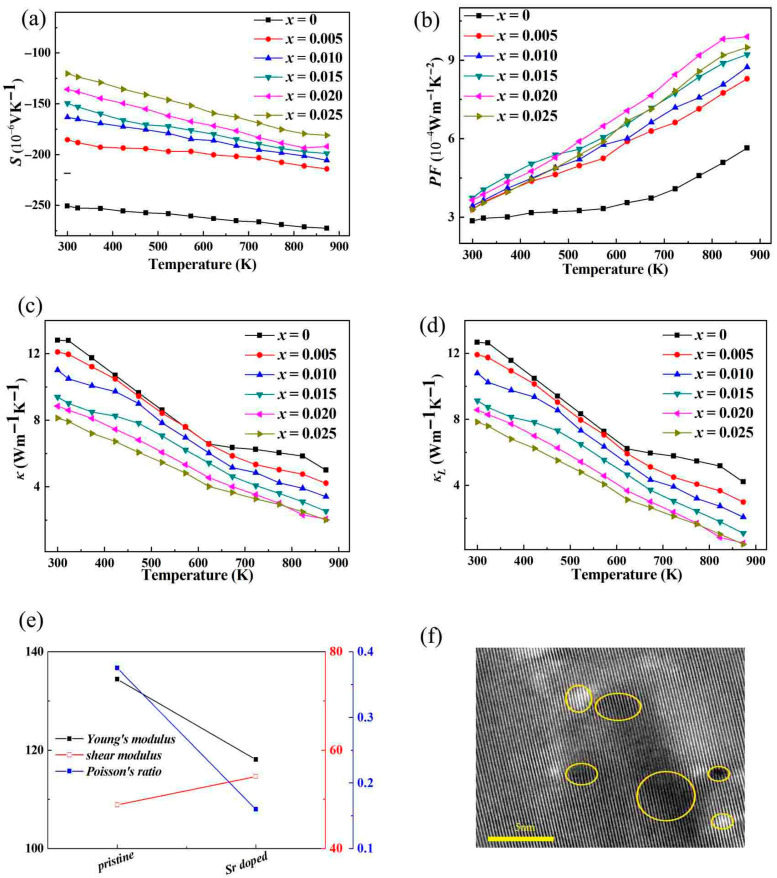
(**a**) The relationship between the Seebeck coefficient (***S***) of the samples and the measured temperature; (**b**) the relationship between the power factor (***PF***) of the samples and the measured temperature; (**c**) the relationship between the thermal conductivity (***κ***) and the measured temperature; (**d**) the lattice thermal conductivity (***κ_L_***) and the measured temperature; (**e**) the elastic constants calculated from CASTEP for ZnO and Sr-doped ZnO; (**f**) the transmission electron microscopy testing results for Sr-doped ZnO(the yellow circles in the TEM image indicate the microdefects induced by Sr doping).

Figure 5a shows the Seebeck coefficients of the Zn_1−*x*_Sr*_x_*O (*x* = 0, 0.005, 0.010, 0.015, 0.020, 0.025) series polycrystalline samples at 300–873 K. The Seebeck coefficients of all samples are negative, corresponding to the N-type electric conduction of ZnO. The absolute value of the thermoelectric potential of the undoped sample decreases with the increase in temperature, while the absolute value of the thermoelectric potential of the doped sample decreases and increases with the increase in temperature. Comparing samples doped with different Sr contents, the higher the doping amount, the smaller the absolute value of thermoelectric potential. This is due to the mutual constraint between the thermoelectric potential ***S*** and the carrier concentration ***n***, as shown in Equation (1):

(1) $$S=\pm \frac{k_{B}}{e}\left[\gamma +2+\ln\frac{2{\left(2\pi m*k_{B}T\right)}^{3/2}}{h^{3}n}\right]$$

The larger the ***n***, the lower the ***S***, while Sr doping can effectively increase the carrier concentration of ZnO [39], resulting in a decrease in the absolute value of the thermoelectric potential of the doped sample. At ***x*** = 0.025, the carrier concentration reaches its maximum and the absolute value of the thermoelectric potential reaches its minimum. The Seebeck coefficient, which determines the efficiency of converting temperature differences into electrical voltage in thermoelectric materials, is related to the energy-dependent distribution of charge carriers. When a temperature gradient is applied, electrons with different energies will respond differently. If the energy-filtering is optimized, it is possible to increase the Seebeck coefficient. However, this is a delicate balance, as the increase in carrier concentration due to the density of states can also have a counter-effect on the Seebeck coefficient. In general, an increase in carrier concentration can lead to a decrease in the Seebeck coefficient. But in the case of Sr-doped ZnO, the complex interplay between the density of states, the change in the energy-filtering effect, and the increase in carrier concentration needs to be carefully analyzed. The power factor (***PF***) at 300–870 K of Zn_1−*x*_Sr*_x_*O (*x* = 0, 0.005, 0.010, 0.015, 0.020, 0.025) polycrystalline samples is calculated from ***σ***-***T.*** The ***S***-***T*** curve is shown in Figure 5b. The power factor of all samples increases with increasing temperature; the maximum power factor at room temperature for ***x*** = 0.015 is approximately ~3.741 μWcm^−1^K^−2^, and the maximum power factor at 873 K for ***x*** = 0.020 reaches ~9.901 μWcm^−1^K^−2^, which is ~76% higher than that of the pristine ZnO.

As shown in Figure 5c, the thermal conductivity (***κ***) of the doped sample decreased with increasing temperature, and the thermal conductivity reached ~12.81 Wm^−1^K^−1^ near room temperature and ~5.01 Wm^−1^K^−1^ at 873 K. The thermal conductivity of doped samples decreases compared to undoped ZnO samples throughout the entire testing temperature range. Although Sr doping improves the electrical conductivity and corresponding electronic thermal conductivity of the sample (***κ*_E_** *=* ***LσT***, ***L*** is Lorentz constant), the proportion of electronic thermal conductivity in the total thermal conductivity is still relatively small, and the trend of lattice thermal conductivity (***κ*_L_**) of the doped sample as shown in Figure 5d is similar to that of thermal conductivity. There are three possible reasons for the decrease in the lattice thermal conductivity of Sr-doped samples. First, Sr has a certain difference in ionic radius (~1.18 Å), electronegativity (~0.95) and mass (~87.62) from Zn ions (~0.74 Å, ~1.65, ~65.41) and causes lattice distortion such as Zn-O bond shortening, as shown in Table 1. The lattice defects, such as point defect scattering, introduced by Sr doping would cause the anharmonic lattice vibration to strengthen [40]. The distribution of black and white areas in the lattice TEM testing results of Sr-doped samples as shown in Figure 5f also verifies this point. The yellow circles in the TEM image indicate the microdefects induced by Sr doping, which have a positive effect on lattice vibrations, phonon scattering, and the corresponding reduction in lattice thermal conductivity. The bright and dark regions may directly reflect the distribution of atomic columns—areas with high atomic density (e.g., dense Zn-O atomic columns) appear as dark spots due to electron phase differences, while regions with sparse atoms or interstitial spaces appear brighter. When Sr atoms substitute Zn atoms, if local ordered structures form, they may manifest as spots with varying darkness in atomic-scale images. Secondly, after Sr doping, Young’s modulus decreases, as shown in Figure 5e, which also could result in a decrease in lattice thermal conductivity. Thirdly, Sr (~87.62) is a “heavier” element relative to Zn (~65.41), and the introduction of heavy elements also benefits the reduction in lattice thermal conductivity [41]. The reduction in lattice thermal conductivity in Sr-doped ZnO samples has far-reaching implications for its thermoelectric performance. The decrease in Young’s modulus after Sr doping also plays a significant role. The introduction of the “heavier” Sr element has a unique impact on phonon behavior. Heavier atoms like Sr introduce a new set of vibrational modes. These new modes have lower frequencies compared to the vibrational modes of the lighter Zn atoms. The presence of these lower-frequency modes can lead to a more complex phonon–phonon interaction spectrum. The lower-frequency phonons can couple with the higher-frequency phonons in the lattice. This coupling can cause additional scattering events, as the different-frequency phonons interact in ways that disrupt the smooth flow of heat. Moreover, the heavier Sr atoms can act as mass-defect scattering centers for phonons. When electron–phonon scattering increases, it can serve as an additional heat dissipation mechanism, which may reduce thermal conductivity. However, the specific change in thermal conductivity depends on the relative strength between electron–phonon scattering and other heat transport mechanisms such as lattice vibrations (phonons). As mentioned earlier, the shortening of the Zn-O bond length after Sr doping also has multiple implications; a shorter bond length generally indicates a stronger chemical bond. This stronger bond can affect the vibrational properties of the lattice. With a stronger Zn-O bond, the phonon frequencies may change. The change in phonon frequencies can lead to a modification of the phonon dispersion relation. A modified phonon dispersion relation can in turn affect the phonon mean free path. If the phonon mean free path decreases, it means that phonons will travel shorter distances before scattering, which can reduce the thermal conductivity. On the other hand, the stronger bond can also make the lattice more rigid, which may increase the speed of sound in the material. An increase in the speed of sound can potentially increase the phonon group velocity and thus increase the thermal conductivity. So, the net effect of the shorter Zn-O bond on thermal conductivity is a complex outcome of these competing factors. The role of Sr in the ZnO lattice is also interesting from a chemical stability perspective. Although Sr has a larger ionic radius than Zn, its incorporation into the ZnO lattice, despite causing lattice expansion, seems to be relatively stable. This stability is important for the long-term performance of the material in various applications. If the Sr-doped ZnO is to be used in high-temperature or harsh-environment applications, the stability of the lattice and the chemical bonds becomes crucial. The strong atomic hybridization between Sr, Zn, and O atoms, which contributes to the increase in the density of states, also plays a role in maintaining the stability of the lattice.

**Figure 6:**
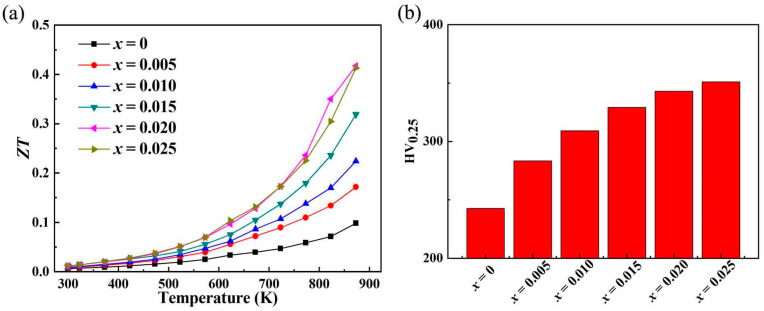
(**a**) The relationship between the ***ZT*** of the Sr-doped ZnO samples and the measured temperature; (**b**) the Vickers hardness testing results for ZnO and Sr-doped ZnO.

This study achieves the dual effect of increasing the expected electrical conductivity while further reducing the thermal conductivity through Sr doping. Figure 6a shows the chart diagram of ***ZT*** of ZnO samples changing with temperature. Due to the substantial increase in electrical conductivity and the decrease in thermal conductivity, the ***ZT*** value of the sample after Sr doping significantly increased, reaching ~0.418 at 873 K, which is ~4.2 times the highest ***ZT*** of the undoped ZnO sample, indicating that Sr doping can be achieved through powder metallurgy and discharge plasmon sintering, which greatly improves the thermoelectric properties of zinc oxide. The ZT value of this study is higher than ~0.09 for Ni-doped ZnO [42], ~0.057 for Al/Ni-doped ZnO [43], and ~0.17 for Ga/In-doped ZnO [44] and lower than Ga-doped ZnO [45]. The average ZT value of the optimal doped sample reaches ~0.17. When preparing ZnO thermoelectric devices (generator or refrigeration devices), small square particle materials may break, so improving the mechanical properties of ZnO materials is also very important. The Vickers hardness test results of ZnO before and after Sr doping are shown in Figure 6b. The Vickers hardness of ZnO reached ~242.7 HV, which is higher than that of alloy thermoelectric materials such as bismuth telluride [46,47]. After Sr doping, the Vickers hardness further increases, reaching ~351.1 HV at most, an increase of 45% compared to the pristine ZnO. The substantial increase in the ZT value of Sr-doped ZnO is a significant achievement. This improvement can be attributed to the delicate balance struck between enhancing electrical conductivity and reducing thermal conductivity. The enhanced electrical conductivity, driven by the increase in carrier concentration due to Sr-induced changes in the electronic structure, allows for more efficient charge transport. This means that a greater number of electrons are available to carry the electrical current, reducing the electrical resistance of the material. On the other hand, the reduction in thermal conductivity, particularly lattice thermal conductivity, is equally crucial. The lattice distortion caused by Sr doping disrupts the regular lattice vibrations, increasing phonon scattering. Phonons are the carriers of heat in the lattice, and more scattering events lead to a decrease in their ability to transport heat efficiently. As a result, less heat is conducted through the material, which is highly beneficial for thermoelectric applications. In a thermoelectric generator, for example, a lower thermal conductivity ensures that more of the heat energy is converted into electrical energy rather than being wasted as heat flow through the material. The improvement in mechanical properties, as evidenced by the significant increase in Vickers hardness, holds far-reaching implications. Undoped ZnO already has a relatively high Vickers hardness, which is even higher than that of some alloy thermoelectric materials such as bismuth telluride, indicating its potential for applications requiring mechanical strength. After Sr doping, the hardness further increases to ~351.1 HV, making Sr-doped ZnO even more appealing. This enhanced mechanical robustness can prevent small square particle materials from breaking during the preparation of ZnO thermoelectric devices. In practical applications, such as thermoelectric generators that may be subjected to mechanical stress during operation or installation, the improved hardness can ensure the long-term stability and reliability of the devices.

## 4. Conclusions

Here, Sr-doped ZnO samples were prepared through room-temperature powder making and high-temperature block making in order to improve thermoelectric performances. The results show that Sr doping could cause the Zn-O bond to become shorter; in addition, the hybridization between Zn and O atoms would become stronger, and the Sr atom itself would modify the density of states near the Fermi level, which could significantly increase the carrier concentration, electrical conductivity, and the corresponding power factor. Sr doping could cause lattice distortion, enhance phonon scattering effects, and decrease the lattice thermal conductivity and thermal conductivity. Sr doping can achieve the effect of improving electrical transport performance and decreasing thermal transport performance. In summary, the doping of Sr into ZnO has demonstrated remarkable potential for enhancing the material’s thermoelectric and mechanical properties. The unique atomic characteristics of Sr play a pivotal role in these improvements. By causing the shortening of the Zn-O bond and strengthening the hybridization between Zn and O atoms, Sr effectively modulates the electronic structure of ZnO. This modulation not only boosts the carrier concentration and electrical conductivity but also significantly improves the power factor, which is crucial for efficient thermoelectric energy conversion. Simultaneously, Sr doping induces lattice distortion. This distortion could enhance the phonon scattering, resulting in a substantial reduction in lattice thermal conductivity and overall thermal conductivity. This combination of enhanced electrical transport and reduced thermal transport is highly desirable for thermoelectric applications, as it maximizes the conversion of heat into electricity. The significant increase in the ZT value, reaching ~0.418 at 873 K, which is a staggering ~4.2 times that of the undoped sample, clearly showcases the effectiveness of Sr doping in enhancing thermoelectric performance. Moreover, the increase in Vickers hardness to ~351.1 HV, a 45% improvement over pristine ZnO, indicates that Sr doping can also enhance the material’s mechanical strength. This added mechanical robustness further broadens the potential applications of Sr-doped ZnO, making it suitable for use in environments where both good thermoelectric performance and mechanical integrity are required. Overall, Sr-doped ZnO emerges as a promising material for various energy-related and mechanical applications.

## Figures and Tables

**Table 1:** The crystallography data results calculated by Castep module of Materials Studio.

	Pristine ZnO	Sr Doped ZnO
Lattice constant ***a***	~3.249 Å	~3.250 Å
Lattice constant ***c***	~5.205 Å	~5.211 Å
Length of bond of O-Zn (I)	~2.006 Å	~1.818 Å–~1.862 Å
Length of bond of O-Zn (II)	~2.012 Å	~1.789 Å–~1.854 Å
Length of bond of O-Sr (I)	-	~2.018 Å
Length of bond of O-Sr (II)	-	~2.136 Å
Angle of O-Zn-O	~108.661°	~106.915°–~111.157°
Angle of Zn-O-Zn	~110.269°	~107.101°–~117.271°
Angle of O-Sr-O	-	~111.757°
Angle of Zn-O-Sr	-	~104.961°

## Data Availability

The raw/processed data required to reproduce these findings cannot be shared at this time as the data also forms part of an ongoing study.
